# An lncRNA Model for Predicting the Prognosis of Hepatocellular Carcinoma Patients and ceRNA Mechanism

**DOI:** 10.3389/fmolb.2021.749313

**Published:** 2021-11-12

**Authors:** Hao Zhang, Renzheng Liu, Lin Sun, Xiao Hu

**Affiliations:** ^1^ Department of Hepatobiliary Pancreatic Surgery, The Affiliated Hospital of Qingdao University, Qingdao, China; ^2^ Department of ICU, The Affiliated Hospital of Qingdao University, Qingdao, China

**Keywords:** lncRNA, model, prognosis, hepatocellular carcinoma, ceRNA

## Abstract

Liver cancer is a highly malignant tumor. Notably, recent studies have found that long non-coding RNAs (lncRNAs) play a prominent role in the prognosis of patients with liver cancer. Herein, we attempted to construct an lncRNA model to accurately predict the survival rate in liver cancer. Based on The Cancer Genome Atlas (TCGA) database, we first identified 1066 lncRNAs with differential expression. The patient data obtained from TCGA were divided into the experimental group and the verification group. According to the difference in lncRNAs, we used single-factor and multi-factor Cox regression to select the genes needed to build the model in the experimental group, which were verified in the verification group. The results showed that the model could accurately predict the survival rate of patients in the high and low risk groups. The reliability of the model was also confirmed by the area under the receiver operating characteristic curve. Our model is significantly correlated with different clinicopathological features. Finally, we built a ceRNA network based on lncRNAs, which was used to display miRNAs and mRNAs related to lncRNAs. In summary, we constructed an lncRNA model to predict the survival rate of patients with hepatocellular carcinoma.

## Introduction

Hepatocellular carcinoma (HCC) is the most common type of primary liver cancer, with high mortality (Farazi and DePinho, 2006). Despite several available treatment methods for HCC, patients in different stages require different treatment measures and the long-term survival remains poor. At present, the staging and grading of HCC is not comprehensive (Zhu et al., 2011), which makes assessment of prognosis difficult. Therefore, it is important to identify predictive indicators and develop a prognostic model for patients with HCC.

As the biological functions of long non-coding RNAs (lncRNAs) have been gradually uncovered, they have become a research hotspot. The function of lncRNAs can be divided into two types: regulating the local chromatin structure and/or gene expression of cis vs. trans, and those leaving the transcription site and performing cell function with trans transcripts (Gil and Ulitsky, 2020). After the role of lncRNAs in regulating tumorigenesis and oncoprotein expression was established, lncRNAs were also found to alter the prognosis of numerous human cancers. Various studies have shown the effect of lncRNAs on the survival of patients with HCC. However, there is no effective prognostic model to evaluate the prognosis and guide the treatment of HCC. The Cancer Genome Atlas (TCGA) database has abundant transcriptome expression profiles of different cancers. Bioinformatics facilitates screening of biomarkers and constructing prediction models using public databases. Through the analysis of gene transcription level using TCGA and bioinformatics, this study found new molecular markers related to HCC survival and established a prognosis model of lncRNAs in HCC. Cox regression analysis, and least absolute shrinkage and selection operator were implemented to validate the lncRNAs associated with prognosis. The power of the prognostic model including four lncRNAs was tested by the receiver operating characteristic (ROC) curve. Statistics and graphic visualization were completed in R software.

Next, we used miRcode database to validate LINC00462 and find the downstream miRNAs. We used miRDB, miRTarBase, and TargetScan databases to find miRNAs targeting specific genes. Finally, we used the Cytoscape to structure the ceRNA network.

## Materials and Methods

### Clinical Samples and Ethics Statement

The ethics committee of the Affiliated Hospital of Qingdao University approved this research. This study included 10 HCC patients (5 males and 5 females), who were diagnosed by pathology, between June 2018 and June 2019, at the Affiliated Hospital of Qingdao University (Shandong, China). The age range was 50–80 years. Tissues were sliced into small sections and stored at −80°C. Corresponding adjacent non-cancerous tissues (ANT) and cancerous tissues were simultaneously collected. The hospital’s Institutional Review Board strictly adhered to the Declaration of Helsinki protocol for approving the study. Written informed consent was signed by each patient. The institutional approval number for human studies is QYFY ZWLL 26019.

### Gene Expression Profiles of HCC Patients

The expression data of lncRNAs, miRNAs, and mRNAs, as well as clinical features were retrieved from TCGA (https://portal.gdc.cancer.gov/). We downloaded the annotation file of transcriptome from Ensembl online database (https://asia.ensembl.org/index.html) and used it to distinguish lncRNAs and miRNAs. There are not copyright issues. Thereafter, we selected differentially expressed genes for this study. The screening of genes was performed in the R software. We used edgeR package to identify differentially expressed genes.

### Definition Of Prognostic Risk Model in the Training Dataset

We randomly divided 342 patients into the training dataset (172) and validation dataset (170). Cox regression analysis, and least absolute shrinkage and selection operator were used to screen prognosis related lncRNAs. Four lncRNAs were screened out in the training dataset. Finally, the following model was established to assess the prognostic risk. Risk score = coefficient_1_ * Expression_1_ + coefficient_2_ * Expression_2_ + coefficient_N_ * Expression_N_. N is the above‐selected lncRNAs, expression is the lncRNA expression in every HCC patient. Cox regression analysis was used to calculate the corresponding lncRNA coefficients in the training dataset. The “survival”, “caret”, “glmnet”, “survminer”, and “survivalROC” packages were used in the analysis process.

### RNA Extraction, Reverse Transcription, and Quantitative Real-Time PCR

We used RNAiso Plus (TaKaRa, Tokyo, Japan) to extract the total RNA from the tissues. The quality of RNA was detected by NanoDrop 2000 spectrophotometer (Thermo Fisher Scientific, Inc.). Next, reverse transcription was performed with T100™ thermal cycler (BIO-RAD, US) using PrimeScript^TM^RT reagent kit and gDNA eraser (TaKaRa, Tokyo, Japan). Quantitative real-time PCR (qPCR) was performed in LightCycler^®^ 96 (Roche, Switzerland) with TB Green®Premix Ex Taq™ Ⅱ (Tli RNase H Plus) (TaKaRa, Tokyo, Japan). Primer sequences are listed in Table 1.

**TABLE 1:** Primer sequences used in the study.

AC016717.2	Forward:5′-GATGCTGATGCTGCCTGTCCATAG-3′
	Reverse:5′-ATGCTGTGCTGTTGGTCTCTGAAG-3′
DDX11-AS1	Forward:5′-CTGGCTACTCTTCCTCCTGG-3′
	Reverse:5′-CAGAGGACATGTGGGAGGTT-3′
LINC00462	Forward:5′-ACTAGGTCCTTCTGGTGTT-3′
	Reverse:5′-GTAAAACTTGCTGCTGATG-3′
ZFPM2-AS1	Forward:5′-GGTGGCACCTGAAATCACAGA-3′
	Reverse:5′-TGCAAGATGACGCTCAGTCG-3′
GAPDH	Forward:5′-TCGGAGTCAACGGATTTGGT-3′
	Reverse:5′-TTCCCGTTCTCAGCCTTGAC-3′

### Statistical Analysis and the Formation of ceRNA Network

According to the median risk score, the patients were divided into the high-risk group and the low-risk group. All the analyses were performed using the R program and software package. The receiver operating characteristic (ROC) curve was used to test the predictive ability of the prognosis model composed of four lncRNAs (Mandrekar, 2010).

We used miRcode (http://www.mircode.org/) (Jeggari et al., 2012), miRDB (http://mirdb.org/) (Chen and Wang, 2020), miRTarBase (http://mirtarbase.mbc.nctu.edu.tw/index.html) (Chou et al., 2018), and TargetScan (http://www.targetscan.org) (Lewis et al., 2005) databases to predict the downstream genes. We used Cytoscape to visualize the ceRNA network.

## Results

### Identification of Differentially Expressed lncRNAs

**FIGURE 1:**
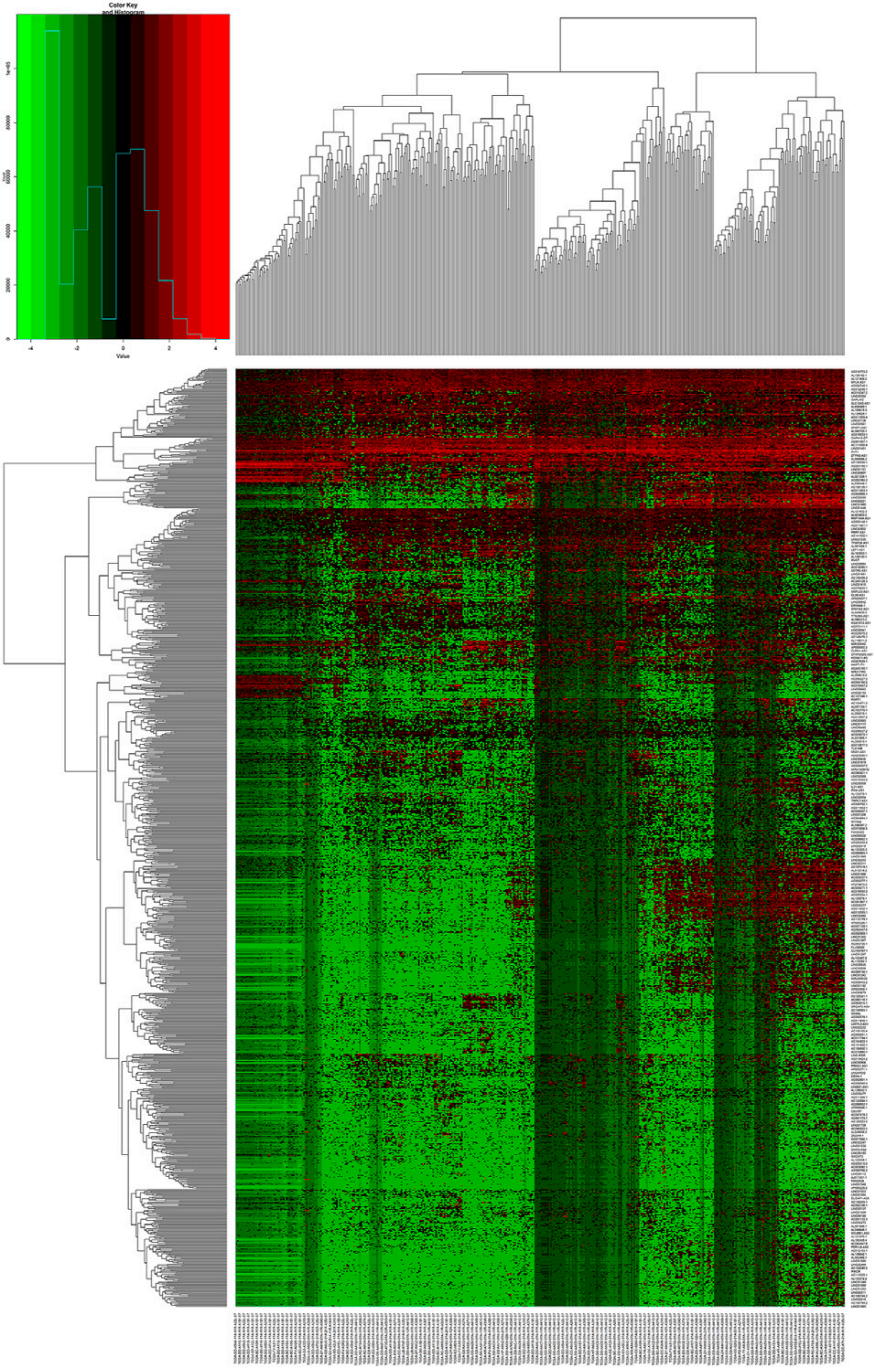
A heat map showing the differential expression of lncRNAs.

We downloaded the related data of 422 patients, 50 normal cases and 372 tumor cases from TCGA database. There were 1066 out of 6739 lncRNAs with logFC >2 and FDR <0.01 in the TCGA dataset, after removing lncRNAs with extremely similar gene expression between HCC and normal patients (Figure 1).

### Identification of Prognostic lncRNAs From the Training Dataset

**FIGURE 2:**
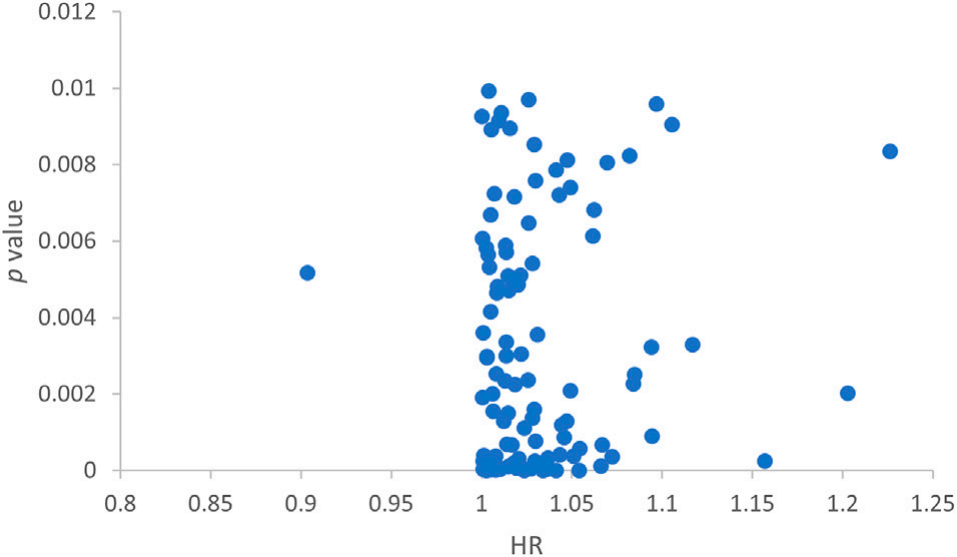
Scatter plot showing lncRNAs screened by single-factor Cox analysis.

We randomly split 342 cancer patients. The training dataset included 172 patients and the test group had 170 patients. We further screened the predicting lncRNAs from the 1066 lncRNAs by univariate Cox analysis. Thereafter, 104 lncRNAs were selected that had an impact on the survival of patients (Figure 2).

### Construction of the Prognostic Model and Checking the Expression of lncRNAs

**FIGURE 3:**
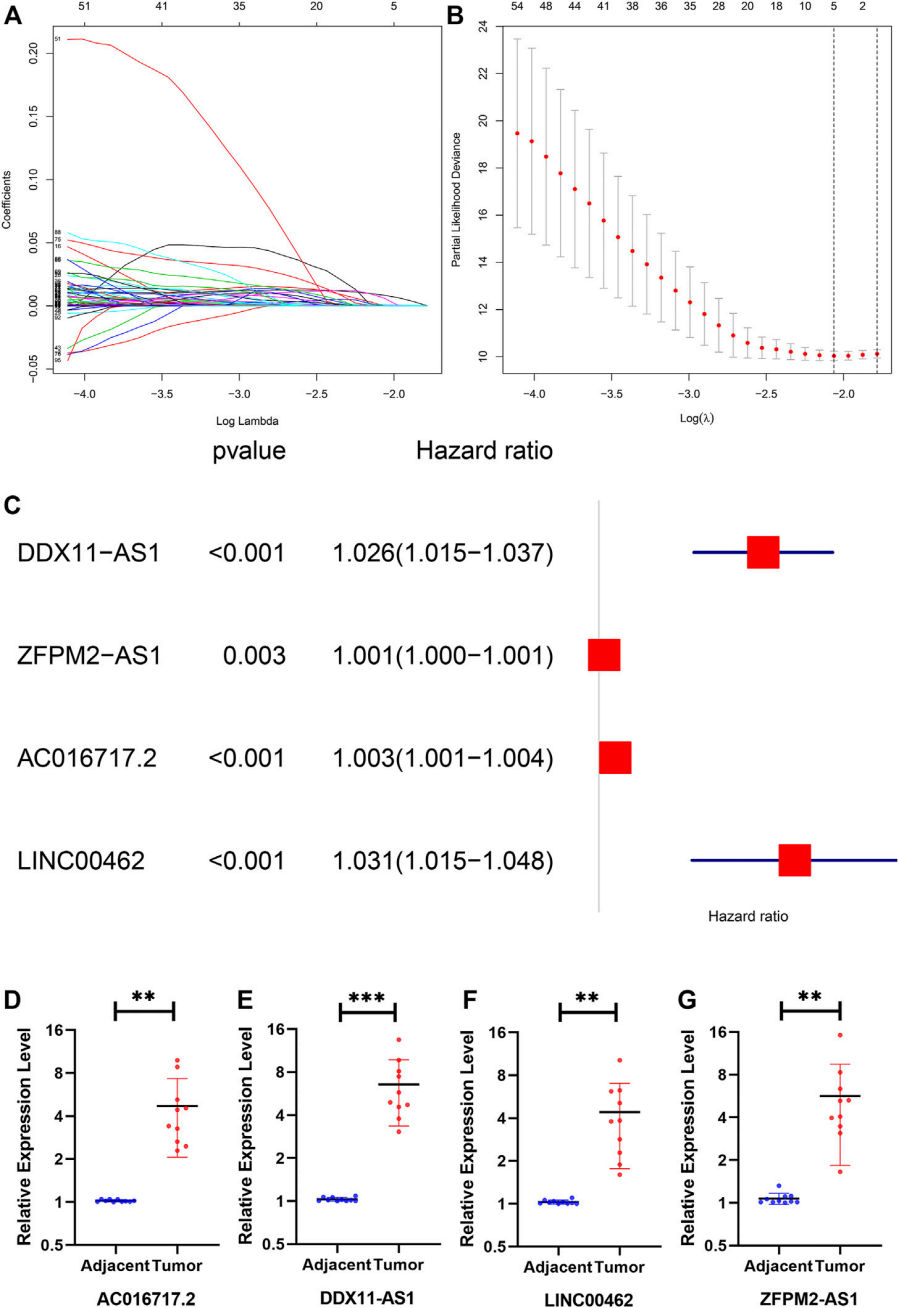
**(A)** The lambda, the cvfit, **(B)** the forest **(C)** the expression of DDX11-AS1, ZFPM2- AS1, AC016717.2, and LINC00462 in tissues. The expression of all genes showed significant differences in 10 tissues.

The number of genes was reduced to 5 lncRNAs by least absolute shrinkage and selection operator (Figure 3A). Finally, four lncRNAs (DDX11-AS1, ZFPM2-AS1, AC016717.2, and LINC00462), which were closely related to patient survival, were selected by multivariate Cox analysis (Figure 3B). A prognostic model composed of the four lncRNAs was established as follows: Risk score = 0.0257 * DDX11-AS1 + 0.0008 * ZFPM2-AS1 + 0.0026 * AC016717.2 + 0.0306 * LINC00462. We verified the transcription levels of these four genes in 10 pairs of human tissue samples (Figure 3C).

### The Model for Survival Prediction

**FIGURE 4:**
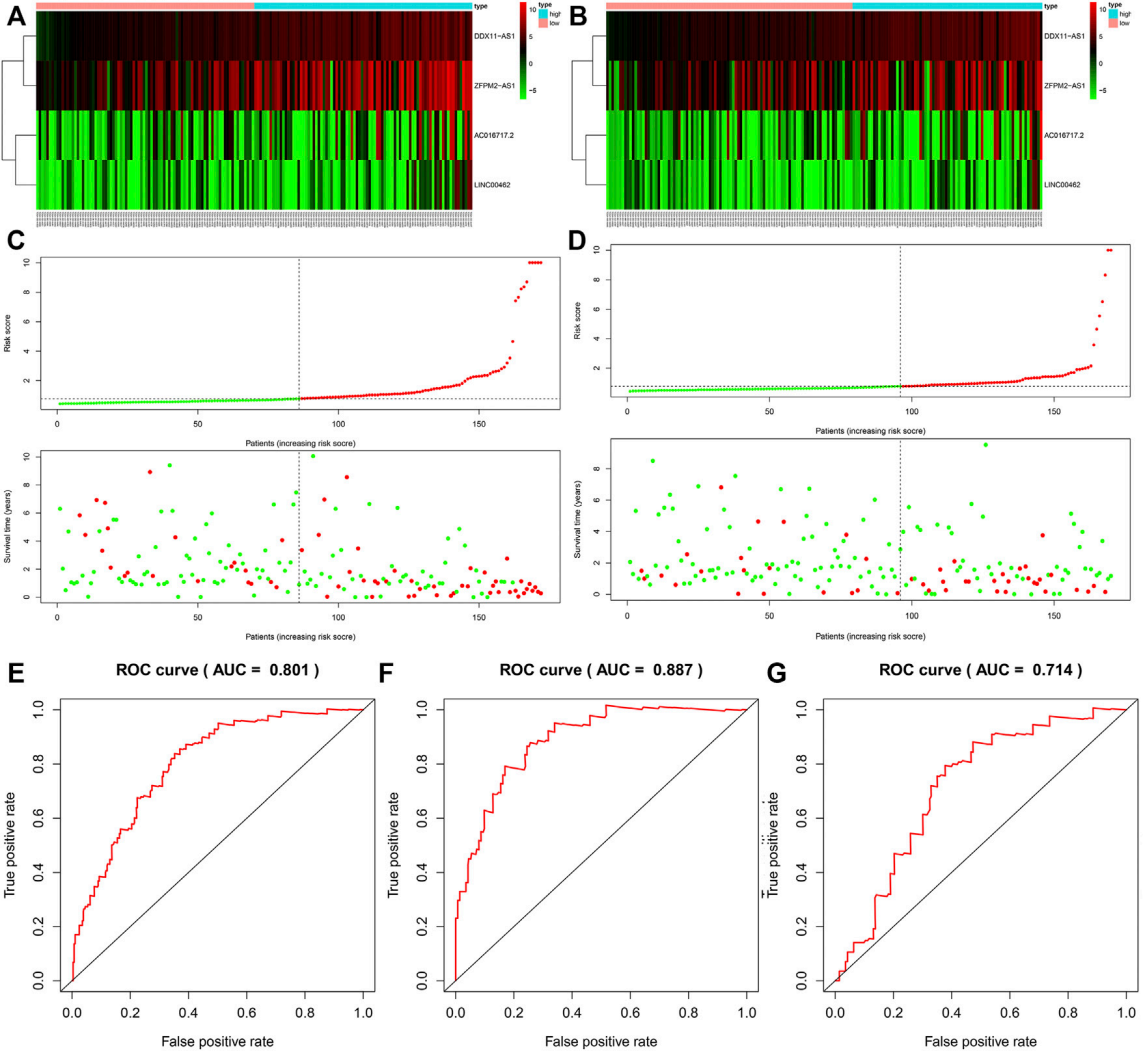
The survival rate of the high-risk group and the low-risk group in the training group **(A)** and the test group **(B)**. The survival status of the training group **(C)** and the test group **(D)**. The ROC curve of the whole group **(E)**, training group **(F),** and the test group **(G)**.

Based on the median risk score, we divided the patients from the training dataset into the high‐risk group (*n* = 86) and the low‐risk group (*n* = 86) and compared the survival of the two risk groups by Kaplan–Meier survival analysis. The survival rate of the high-risk group was significantly lower than that of the low-risk group (Figure 4A). To confirm the predictive power of the prognostic model, we divided the patients in the test dataset into two risk groups according to the median risk score. The Kaplan-Meier curve showed that in the trial cohort, the lifespan of patients in the low-risk group was prominently longer than that in the high-risk group, which was similar to the results obtained from the training dataset (Figure 4B).

The survival status of HCC patients obtained from the training dataset and test dataset is depicted in the plot. The heat map shows the different expression patterns of the four prognosis-related lncRNAs in the low-risk and high-risk populations (Figures 4C,D). Similar to the training dataset, patients with high-risk scores tended to have higher levels of the four lncRNAs than patients with low-risk scores. According to the ROC (AUC) curve, the size of the AUC was related to the prognosis. The ROC curves at 1 year of the whole, training, and validation datasets are shown in Figures 4E–G.

### Prognostic Model and Clinicopathological Features

**FIGURE 5:**
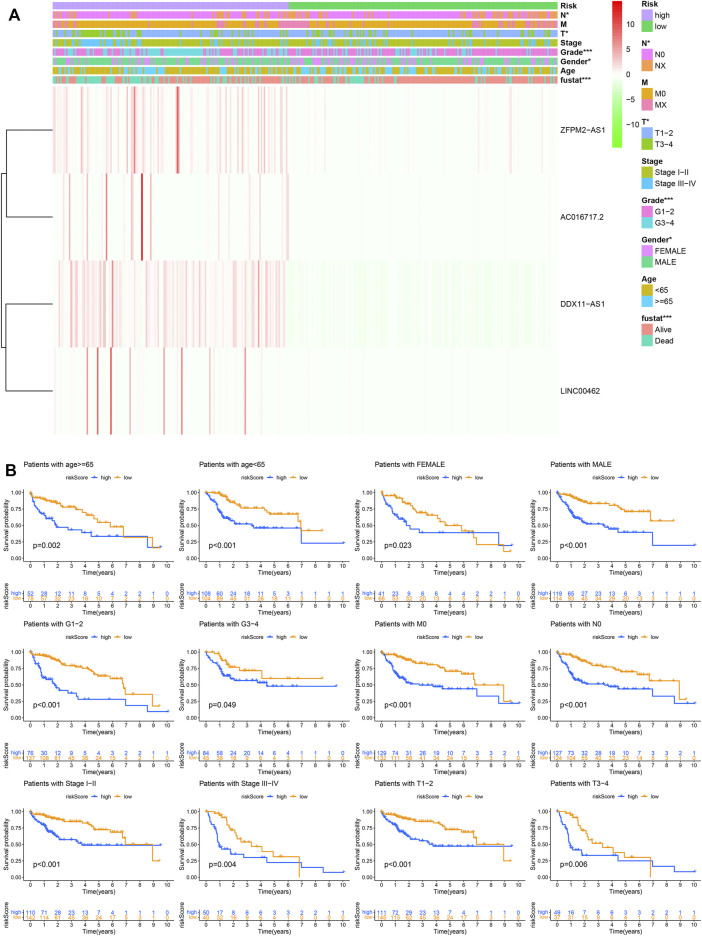
The prognostic model and clinicopathological features. **(A)** The significance of clinicopathological features between high- and low-risk groups. **(B)** Prognostic differences in different groups.

The Chi square test was used to analyze the significance of clinicopathological features between high- and low-risk groups (Figure 5A). In addition, by analyzing the data of patients with different characteristics, we found that the risk score can significantly distinguish the prognostic differences in different age, gender, G stage, M0, N0, stage, and T group (Figure 5B), which significantly improves the prognostic value of our model.

### Construction of the ceRNA Network with the Prognostic lncRNAs

**FIGURE 6:**
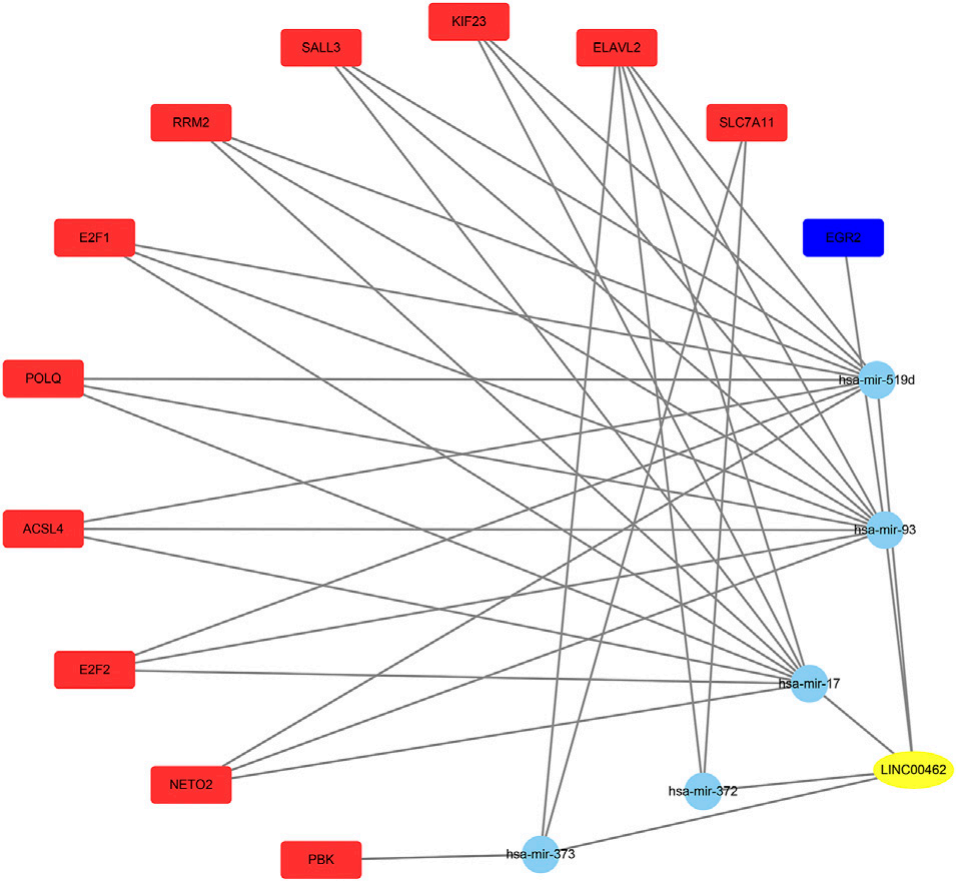
The yellow oval is lncRNA, the blue circles represent miRNAs, the red rectangles represent the up-regulated mRNAs, and the blue rectangle represents the down-regulated mRNA.

Using the TCGA database, 1976 differentially expressed mRNAs were selected from 424 HCC patients (logFC > 2 or logFC < −2,FDR<0.01). From 50 normal samples and 376 tumor samples, 247 differentially expressed miRNAs were selected (logFC > 1 or logFC < −1, FDR < 0.01). We compared DDX11-AS1, ZFPM2-AS1, AC016717.2, and LINC00462 in the miRcode database to identify the downstream miRNAs, and only LINC00462 could be found in the miRcode database. The miRNAs found in the miRcode intersected with the differentially expressed miRNAs in TCGA. The miRDB, miRTarBase, and TargetScan data were combined to predict the downstream mRNAs with the miRNAs obtained in the previous step. The downstream target genes were obtained using the intersection of the differentially expressed mRNAs in the previous TCGA database and the predicted mRNAs in the three databases (Figure 6).

## Discussion

In recent years, with the gradual advancements in the study of lncRNAs, their function in the occurrence and development of HCC has become a research hotspot. Therefore, it is critical to understand the mechanism of action of lncRNAs and comprehensively analyze their clinical significance in patients with liver cancer. The purpose of this study was to establish lncRNA markers related to prognosis of HCC and explore their molecular mechanisms. This study was undertaken to provide a better understanding of the prognosis of patients with liver cancer and facilitate the discovery of biomarkers.

In this study, we downloaded related data of 422 patients from TCGA database and screened out 1066 lncRNAs. We randomly split 342 cancer patients. We further selected the 104 predictive lncRNAs from 1066 lncRNAs by univariate Cox analysis in the training dataset. Finally, four lncRNAs (DDX11-AS1, ZFPM2-AS1, AC016717.2, and LINC00462), which were closely related to patient survival, were selected by least absolute shrinkage and selection operator, and multivariate Cox analysis. A prognostic model or a risk score was constituted with the above lncRNAs. We divided patients from the training dataset into the high‐risk group and the low‐risk group. The survival of patients in the high‐risk group was significantly less than those in the low‐risk group. The survival of patients in the test dataset was similar to the training dataset. Furthermore, ROC curve analysis showed that the lncRNA risk score model had good prediction efficiency in HCC. The AUC value of our model is significantly higher than that of Wu et al. (2020) and Zhou et al. (2021). Finally, we used the miRcode, miRDB, miRTarBase, and TargetScan databases to find the downstream miRNAs and mRNAs, and create the ceRNA network.

DDX11-AS1 was identified as an oncogene in HCC. Silencing the expression of this gene reduced the proliferation, migration, and invasion ability of HCC cells, and the gene could promote the growth of tumors *in vivo* (Wan et al., 2020). DDX11-AS1 was also reported to be related to overall survival (Li et al., 2019). DDX11-AS1 displayed a cancer-promoting role by regulating the expression of related genes directly or indirectly in various malignant tumors, such as osteosarcoma, colorectal cancer, gastric cancer, non-small cell lung cancer, and bladder cancer (Feng et al., 2020). ZFPM2-AS1 regulates the expression of GDF10 through competitive binding to miRNA to promote cell proliferation, migration, invasion, and inhibit apoptosis in HCC (He et al., 2020). As an oncogenic gene, ZFPM2-AS1 plays a role in retinoblastoma (Lyv et al., 2020), breast cancer (Zhao et al., 2020), small cell lung cancer (Yan et al., 2020), cervical cancer (Dai et al., 2020), esophageal squamous cell carcinoma (Sun and Wu, 2020), and other tumors. AC016717.2 is located on chromosome 2 and the gene maps to 226, 563, 230–226, 568, 370 in GRCh37 coordinates. There are few studies on this lncRNA. LINC00462 can significantly increase the invasive ability of HCC cells (Gong et al., 2017). It can also promote pancreatic cancer (Zhou et al., 2018).

In summary, this was the first study to propose a prediction model based on four lncRNAs, in which the effects of DDX11-AS1, ZFPM2-AS1, and lncRNAs on the prognosis of patients with liver cancer have been confirmed. AC016717.2 and LINC00462 are new biomarkers in liver cancer. Although the lncRNAs selected by the predictive model showed good performance, this study only included internal validation results but no external data validation due to resource constraints. Hence, clear evidence with multi-center clinical data is required to validate the results of this study.

## Data Availability

The original contributions presented in the study are included in the article/Supplementary Material. Further inquiries can be directed to the corresponding author.

## Abbreviations

HCC, hepatocellular carcinoma; lncRNAs, long non-coding RNAs; qPCR, quantitative real-time PCR; ROC, receiver operating characteristic; TCGA, The Cancer Genome Atlas.
